# Circulating Tumour Cell Numbers Correlate with Platelet Count and Circulating Lymphocyte Subsets in Men with Advanced Prostate Cancer: Data from the ExPeCT Clinical Trial (CTRIAL-IE 15-21)

**DOI:** 10.3390/cancers13184690

**Published:** 2021-09-18

**Authors:** Brian Hayes, Lauren Brady, Gráinne Sheill, Anne-Marie Baird, Emer Guinan, Bryan Stanfill, Jean Dunne, Dean Holden, Tatjana Vlajnic, Orla Casey, Verena Murphy, John Greene, Emma H. Allott, Juliette Hussey, Fidelma Cahill, Mieke Van Hemelrijck, Nicola Peat, Lorelei A. Mucci, Moya Cunningham, Liam Grogan, Thomas Lynch, Rustom P. Manecksha, John McCaffrey, Dearbhaile M. O’Donnell, Orla Sheils, John J. O’Leary, Sarah Rudman, Ray McDermott, Stephen Finn

**Affiliations:** 1 Department of Histopathology, Cork University Hospital, T12DC4A Cork, Ireland; 2 Department of Pathology, University College Cork, T12DC4A Cork, Ireland; 3 Department of Histopathology and Morbid Anatomy, Trinity Translational Medicine Institute, Trinity College Dublin, D08NHY1 Dublin, Ireland; bradyl4@tcd.ie (L.B.); bairda@tcd.ie (A.-M.B.); john.greene@oncology.ox.ac.uk (J.G.); e.allott@qub.ac.uk (E.H.A.); osheils@tcd.ie (O.S.); olearyjj@tcd.ie (J.J.O.); stephen.finn@tcd.ie (S.F.); 4 Discipline of Physiotherapy, School of Medicine, Trinity College Dublin, D08NHY1 Dublin, Ireland; sheillg@tcd.ie (G.S.); jmhussey@tcd.ie (J.H.); 5 Trinity Centre for Health Sciences, School of Medicine, Trinity College Dublin, D08NHY1 Dublin, Ireland; guinane1@tcd.ie; 6 Pacific Northwest National Laboratory, Richland, WA 99352, USA; bstanfill2003@gmail.com; 7 Department of Immunology, St James’s Hospital, D08NHY1 Dublin, Ireland; jedunne@stjames.ie (J.D.); dholden@stjames.ie (D.H.); 8 Institute of Pathology, University Hospital Basel, 4056 Basel, Switzerland; tatjana.vlajnic@usb.ch; 9 Cancer Trials Ireland, D11KXN4 Dublin, Ireland; caseyorla@gmail.com (O.C.); verena.murphy@cancertrials.ie (V.M.); 10 Centre for Cancer Research and Cell Biology, Queen’s University Belfast, Northern Ireland, Belfast BT9 7AE, UK; 11 King’s College London, School of Cancer and Pharmaceutical Sciences, Translational Oncology and Urology (TOUR), London SE1 9RT, UK; Fidelma.cahill@gstt.nhs.uk (F.C.); mieke.vanhemelrijck@kcl.ac.uk (M.V.H.); 12 Guy’s and St Thomas’ NHS Foundation Trust, London SE1 9RT, UK; nicola.peat@gstt.nhs.uk (N.P.); sarah.rudman@gstt.nhs.uk (S.R.); 13 Department of Epidemiology, Harvard T.H. Chan School of Public Health, Boston, MA 02115, USA; lmucci@hsph.harvard.edu; 14 Department of Radiation Oncology, St Luke’s Hospital, D06HH36 Dublin, Ireland; moya.cunningham@slh.ie; 15 Department of Oncology, Beaumont Hospital, D09V2N0 Dublin, Ireland; liamgrogan@beaumont.ie; 16 Department of Urology, St James’s Hospital, D08NHY1 Dublin, Ireland; tlynch@stjames.ie (T.L.); rustom.manecksha@tcd.ie (R.P.M.); 17 Department of Surgery, Trinity College Dublin, D08NHY1 Dublin, Ireland; 18 Department of Oncology, Mater Misericordiae Hospital, D07AX57 Dublin, Ireland; profjohnmccaffrey@gmail.com; 19 HOPE Directorate, St James’s Hospital, D08NHY1 Dublin, Ireland; dodonnell@stjames.ie; 20 Department of Histopathology, St James’s Hospital, D08NHY1 Dublin, Ireland; 21 Department of Oncology, Tallaght University Hospital, D24NR0A Dublin, Ireland; ray.mcdermott@tuh.ie

**Keywords:** prostate cancer, circulating tumour cells, exercise, flow cytometry, platelets, inflammation, obesity, coagulation

## Abstract

**Simple Summary:**

Cancer cells (CTCs) can be found in the bloodstream in men with advanced prostate cancer. Blood platelets, which normally help the blood to clot, may help the cancer cells to spread throughout the body by preventing the body’s immune system from finding and destroying them while they are in the bloodstream. Blood samples were taken from men with prostate cancer who were involved in the ExPeCT clinical trial, some of whom were taking part in a regular exercise programme. The numbers of CTCs, platelets and immune system cells were counted and compared. Blood samples with more CTCs had higher numbers of platelets and higher numbers of some types of immune system cells. Some differences were also found in men involved in the exercise programme. This study helps to show that CTCs numbers are related to platelet and immune cell numbers in the blood.

**Abstract:**

Interactions between circulating tumour cells (CTCs) and platelets are thought to inhibit natural killer(NK)-cell-induced lysis. We attempted to correlate CTC numbers in men with advanced prostate cancer with platelet counts and circulating lymphocyte numbers. Sixty-one ExPeCT trial participants, divided into overweight/obese and normal weight groups on the basis of a BMI ≥ 25 or <25, were randomized to participate or not in a six-month exercise programme. Blood samples at randomization, and at three and six months, were subjected to ScreenCell filtration, circulating platelet counts were obtained, and flow cytometry was performed on a subset of samples (*n* = 29). CTC count positively correlated with absolute total lymphocyte count (r^2^ = 0.1709, *p* = 0.0258) and NK-cell count (r^2^ = 0.49, *p* < 0.0001). There was also a positive correlation between platelet count and CTC count (r^2^ = 0.094, *p* = 0.0001). Correlation was also demonstrated within the overweight/obese group (*n* = 123, *p* < 0.0001), the non-exercise group (*n* = 79, *p* = 0.001) and blood draw samples lacking platelet cloaking (*n* = 128, *p* < 0.0001). By flow cytometry, blood samples from the exercise group (*n* = 15) had a higher proportion of CD3+ T-lymphocytes (*p* = 0.0003) and lower proportions of B-lymphocytes (*p* = 0.0264) and NK-cells (*p* = 0.015) than the non-exercise group (*n* = 14). These findings suggest that CTCs engage in complex interactions with the coagulation cascade and innate immune system during intravascular transit, and they present an attractive target for directed therapy at a vulnerable stage in metastasis.

## 1. Introduction

Circulating tumour cells (CTCs) are an intermediate stage of metastasis, whereby a cancer can spread from a primary site to set up secondary malignant growths at anatomically distant sites. CTC enumeration may have a prognostic role in advanced prostate cancer (PrCa). A prospective study of 231 men with castration-resistant disease found that more than or equal to five CTCs per 7.5 mL of blood correlated with a poor prognosis when assessed either before or after the initiation of a new line of chemotherapy [1]. Recent studies have further emphasised the negative prognostic impact of numbers of EpCAM-expressing CTCs in metastatic PrCa [2]. CTCs are a potentially useful target for therapy as intravascular cells are vulnerable to the cellular and humoral processes of the innate and adaptive immune systems.

Several different systems exist for CTC enumeration. The FDA-approved system (CellSearch^®^, Menarini Silicon Biosystems, Inc., Huntingdon Valley, PA 19006, USA) for CTC enrichment involves CTC separation from leucocytes by virtue of their affinity for certain specific antibodies, for example EpCAM. The ScreenCell^®^ system (ScreenCell^®^, Paris, France) does not rely on EpCAM expression and instead employs a microporous membrane filter and a vacuum tube to trap large and poorly deformable CTCs on the filter [3]. These can then be subjected to morphological analysis [4], immunohistochemistry [5], or molecular genetic studies [6].

There is evidence that platelets support tumour metastasis by various mechanisms [7,8], including inhibiting CTC killing by the natural killer (NK) cells of the innate immune system [9]. Platelets are involved in arrest of CTCs in the vasculature and through endothelial interactions enable their extravasation. Platelets also secrete various pro-oncogenic factors including PDGF and VEGF, and mediate pro-survival signals in ovarian cancer cells [10]. The interactions between CTCs and platelets are complex, but overall tumour cell-induced platelet aggregation correlates with metastatic potential and may be due to the “cloaking” of tumour cells by adherent platelets. The interaction between CTC cloaking by platelets and their killing by NK-cells is incompletely understood. Thrombocytopaenic mice exhibited a reduced tumour metastatic burden when tumour cells were NK-cell sensitive, and in vitro studies demonstrated reduced NK-cell tumourilytic activity when platelets aggregated around tumour cells [11]. In a later mouse model, deficiency for Gαq, a G-protein critical for platelet activation, markedly decreased experimental and spontaneous metastases [12]. This effect was eliminated in NK-cell deficient mice, further supporting the hypothesis that adherent platelets may obstruct the direct cell-cell contact required for NK-cell killing. CTCs which are coated with platelets have reduced expression of ligands which bind to the NK-cell activating receptor NKG2D [13]. Release of TGFβ by adherent platelets, which downregulates NKG2D [14], may also inhibit NK-cell-tumour interactions. Platelets may enable evasion of NK-cell killing by conferring a “pseudonormal” phenotype on CTCs through high tumour cell surface expression of normal MHC class I antigen [9]. The ExPeCT trial (Exercise, Prostate Cancer and Circulating Tumour Cells) was designed to explore the relationships between platelet cloaking and CTCs in men with advanced PrCa, with particular reference to the impact of obesity and exercise. Regular exercise has well-documented benefits in PrCa, both for primary prevention, amelioration of treatment-related side-effects, and increasingly for improved cancer-specific overall and progression-free survival (reviewed [15]). The primary endpoints of the ExPeCT trial have been published [16]. CTC numbers varied over time, but no significant differences in CTC numbers were found between non-exercise and exercise cohorts, or between overweight participants and those of normal weight. Platelet cloaking was identified in 29.5% of participants. CTC numbers correlated with peripheral blood white cell count, and CTC clusters correlated with PSA levels.

The current study is a secondary analysis from the ExPeCT trial. Its main aim is to determine whether blood samples from ExPeCT trial participants demonstrate a correlation between CTC numbers and platelet counts and circulating lymphocyte subsets, particularly NK-cells, as determined by flow cytometry. A secondary aim is to exploit the design structure of the ExPeCT trial to allow comparisons in these parameters between participants with elevated or normal BMI, and between those who were randomized or not to participate in an organised exercise programme.

## 2. Materials and Methods

### 2.1. ExPeCT Trial Design

The design of the ExPeCT trial (ClinicalTrials.gov identifier NCT02453139, CTRIAL-IE 15-21) has been described elsewhere [17]. In brief, participants with advanced PrCa (metastatic disease confirmed by CT/MRI or bone scan) and no history of radical prostatectomy who were considered capable of safely participating in a six-month exercise programme were recruited at hospitals in Dublin, Ireland, and London, United Kingdom, and randomized to an exercise group or a control group. Subgroup analysis in normal weight and overweight/obese groups was performed based on body mass index (BMI < 25 or ≥25 kg/m^2^). The exercise group participated in a six-month exercise programme, including a weekly group exercise class and a home-based exercise programme. The control group was not given specific exercise advice beyond that considered usual for medical care, and did not participate in the exercise programme. Blood samples were taken for CTC enumeration, full blood count and platelet count at the time of recruitment (T0) and after three (T3) and six (T6) months.

### 2.2. Preparation and Analysis of ScreenCell^®^ Filters

Blood samples were acquired from each participant for ScreenCell^®^ filtration in 3 mL K2-EDTA tubes (Grenier Bio, Monroe NC USA) using a 23-gauge, 3/4 inch butterfly needle. Up to four tubes were acquired at each blood draw (yielding a total of up to 12 mL on each occasion) and processed with ScreenCell^®^ Cyto filtration units within four hours according to previously published protocols [16]. Filters were stained with May–-Grunwald–Giemsa (MGG), stored at 4 °C and examined by a pathologist (BH) using an Olympus BX41 light microscope (Olympus Corporation, Tokyo, Japan) with 10× and 40× Olympus Plan Fluorite objective lenses and a 20× Olympus Plan Apo objective lens. Filters were screened at 20× with the condenser in situ to enumerate CTCs. The presence (and number) of CTCs and the presence or absence of platelet cloaking were recorded per filter and per blood draw using a Microsoft Excel spreadsheet (Microsoft, Redmond WA USA). The mean number of CTCs per filter was calculated for each blood draw episode. The microscopist was blinded as to whether the filter was from an exercise- or control-group participant, but not as to whether it was derived from a T0, T3 or T6 blood draw.

As described previously [16], CTCs were defined as cells in the same plane of focus as filter pores whose nucleus was at least twice the diameter of a filter pore, dark blue/purple in colour, generally of uniform staining intensity and with an outline which was well-defined around its entire circumference. Many CTCs were centred on filtration pores and had markedly irregular nuclear contours, but these were not considered definitional features. The presence of cytoplasm was not required to define a CTC as many CTCs lacked any cytoplasm and were considered “bare nuclei”.

Platelet cloaking of an individual CTC was defined as the presence of at least one platelet in direct contact with the edge of the CTC. In order to distinguish true platelet cloaking from procedure-related blood clot, platelet cloaking was only confirmed when a cloaked CTC was identified away from any areas of fibrin/platelet clot at the surface of the filter. Dense three-dimensional clusters of platelets were only considered to represent platelet cloaking if a definite CTC could be identified within.

### 2.3. Flow Cytometry

Blood samples for flow cytometry were taken in K2-EDTA tubes at each timepoint from a subset of Dublin-based participants. The specimens were transported promptly to the laboratory and analysed immediately using a BD Multitest 6-color TBNK reagent (BD Biosciences, San Jose, CA, USA), which contains FITC-labeled CD3 (clone SK7); PE-labeled CD16 (clone B73.117-19); CD56 (clone NCAM16.2;20); PerCP-Cy™5.5–labeled CD45 (clone 2D1 {HLe-1};21); PE-Cy™7–labeled CD4 (clone SK3;22-24); APC-labeled CD19 (clone SJ25C1;25); APC-Cy7–labeled CD8 (clone SK1.22,23). A BD FACSCanto II flow cytometer (BD Biosciences) was employed and data analysed using BS FACSCanto Clinical software (BD Biosciences).

### 2.4. Statistical Methods

GraphPad InStat Version 3.10 (GraphPad Software, Inc, San Diego, CA, USA) was used for statistical analysis. Contingency tables with two rows and columns were analysed using Fisher’s exact test with a two-tailed *p*-value. Larger contingency tables were examined using the Chi square test for independence, with Chi square test for trend reported in cases with *p* < 0.05. For comparison of means between two groups the unpaired *t*-test with a two-tailed *p*-value was employed when the standard deviations of the groups were not significantly different and the data were distributed in a Gaussian fashion as determined by the Kolmogorov–Mirnov test. The Mann–Whitney test with a two-tailed *p*-value was used for data that failed the Kolmogorov–Mirnov normality test. For a comparison of means in more than two groups, the Kruskall–Wallis test (nonparametric analysis of variance, ANOVA) was employed when Bartlett’s test determined that there was a significant difference between the standard deviations of the groups. When *p* < 0.05 by the Kruskall–Wallis test, Dunn’s multiple comparison test was undertaken to determine the level of significance of differences between individual groups. For comparison of means in more than two groups with Gaussian distribution and no significant difference in standard deviations, a one-way analysis of variance (ANOVA) was undertaken. When the values in each row were matched (for example, assessment of a given variable across the three timepoints), repeated measures ANOVA were performed, and both the F-statistic and *p*-value were reported. This technique required the exclusion of any records that lacked complete data, such as a lack of blood draw at a given timepoint for an individual participant. For distributions not assumed to be normally distributed, or when the numbers of samples were low, the Friedman test (nonparametric repeated measures ANOVA) was employed, with reporting of the two-tailed *p*-value and the Friedman statistic (Fr).

Regression analysis was employed to test for the presence of a relationship between a dependent continuous variable (*y*) and a single explanatory (*x*) variable (linear regression), or multiple explanatory variables (multiple regression). For simple linear regression, GraphPad was used to find the line of best fit through the data by identifying the regression coefficient that minimised the total error of the model. The mean square error was calculated by measuring the distance of the observed *y*-values from the predicted *y*-values at each value of *x*, squaring each of these distances and calculating the mean of each of the squared distances. Goodness of fit was expressed as r^2^, which quantified the proportion of variance in the dependent variable which could be explained by variation in the explanatory variable. A two-tailed *p*-value was calculated, with *p* < 0.05 accepted as the threshold of statistical significance.

In multiple regression analysis a model was constructed defining a single dependent variable as a function of several independent variables. Having checked for multicollinearity, regression coefficients were calculated for each parameter using the least-squares method, with standard errors and upper and lower 95% confidence intervals derived from the t ratio. A *p*-value was calculated for each parameter, derived from the t ratio and the number of degrees of freedom, expressing the probability that the observed impact of the corresponding variable on the model was due to chance. Variables that had parameters with *p*-values less than 0.05 were considered not to explain independently the variance in the dependent variable. R^2^ was calculated, representing the fraction of all variance in the dependent variable explained by the model, and adjusted R^2^ corrected for the number of explanatory variables in the model. An F-test was used to calculate an overall *p* value for the multiple regression which tested the null hypothesis that a model with no independent variables would fit the data as well as this model. Statistical significance was accepted as *p* < 0.05.

## 3. Results

### 3.1. CTCs Were Identified in Almost 95% of ScreenCell^®^ Filters

In all, 61 participants were randomized to the exercise or control group and had at least an initial (T0) blood sample taken for assessment. The clinical and pathological features of the participants and their diagnostic biopsies were reported previously [16]. Eleven participants (18%) had a normal BMI (<25 kg/m^2^). The remaining 50 participants (82%) comprised the overweight/obese group. Several participants withdrew from the study or died between T0 and T3 (*n* = 8) or between T3 and T6 (*n* = 2). One further participant was unavailable for blood sampling at T3, and two at T6. Overall 598 ScreenCell^®^ filters were prepared from 162 blood draws from the 61 participants—their features are summarised in Table 1. The mean number of filters examined per blood draw was 3.7 (range 1–7, median 4). Variation of CTC numbers and other features across timepoints, and comparison between groups, have been reported previously [16].

### 3.2. Flow Cytometry-Derived Lymphocyte Populations

Ten trial participants had blood drawn for flow cytometric immunophenotyping of circulating lymphoid subsets at each timepoint (5 exercise group, 5 control group; 8 overweight/obese, 2 normal BMI). One control-group, overweight/obese-group participant did not have blood drawn at the T3 timepoint, so the total number of blood samples subjected to flow cytometry was 29.

**Figure A1:**
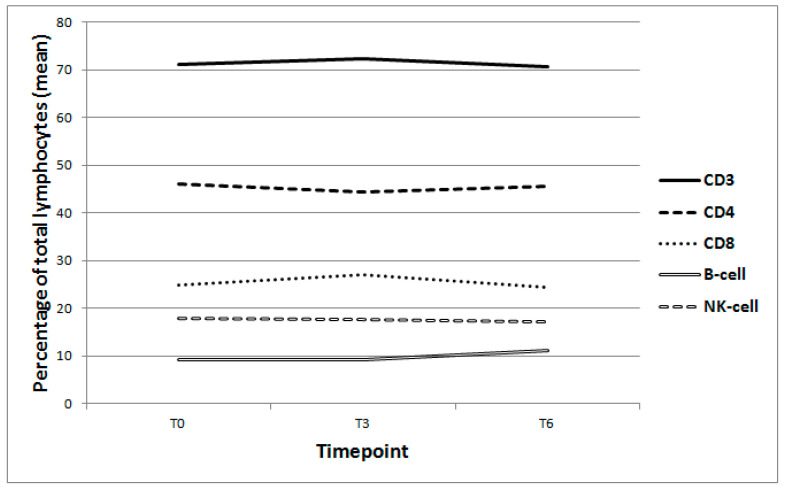
Comparison of mean lymphocyte subset percentages at each timepoint (T0, T3, T6) in participants for whom full data were available (*n* = 9). CD3 *p* = 0.9131 (Friedman test). CD4 *p* = 0.8485; CD8 *p* = 0.1814; B-cell *p* = 0.5109; NK-cell *p* = 0.9466 (Repeated measures ANOVA).

**Figure A2:**
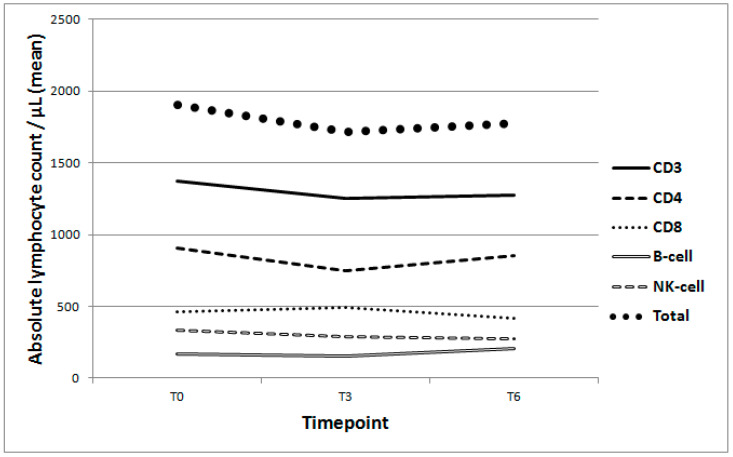
Comparison of mean absolute lymphocyte counts per μL at each timepoint (T0, T3, T6) in participants for whom full data were available (*n* = 9). CD3 *p* = 0.569 (Friedman test). CD4 *p* = 0.6801 (Repeated measures ANOVA). CD8 *p* = 0.6854 (Friedman test). B-Cell *p* = 0.8716 (Repeated measures ANOVA). NK-Cell *p* = 0.898 (Ordinary ANOVA). Total lymphocytes *p* = 0.9712 (Friedman test).

Across all groups and timepoints, CD3+ T-lymphocytes constituted a mean of 71.3% of lymphoid cells in the blood samples. CD4+ and CD8+ cells constituted means of 45.3 and 25.5%, respectively. Smaller proportions of lymphocytes were CD19+ B-lymphocytes (9.9%) or CD56+/CD16+ NK-cells (17.5%). The mean absolute total lymphocyte count was 1800.9/μL, including means of 1304.2 CD3+ T-lymphocytes, 838.5 CD4+ T-lymphocytes, 455.6 CD8+ T-lymphocytes, 174.6 B-lymphocytes, and 300.3 NK-cells. Variation of lymphoid cell populations per timepoint are presented in Appendix A (including Table A1, Figure A1 and Figure A2).

### 3.3. Total Lymphocyte and Absolute NK-Cell Counts Correlate with CTC Numbers

**Figure 1:**
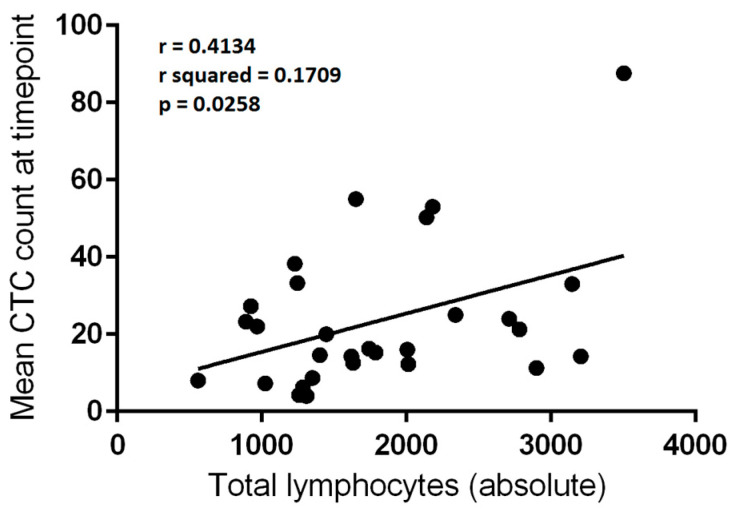
Correlation between absolute total lymphocyte count (per μL) and mean circulating tumour cell (CTC) count per 3 mL of blood at a given blood draw (*n* = 29). Linear regression, r = 0.4134; r^2^ = 0.1709; *p* = 0.0258.

**Figure 2:**
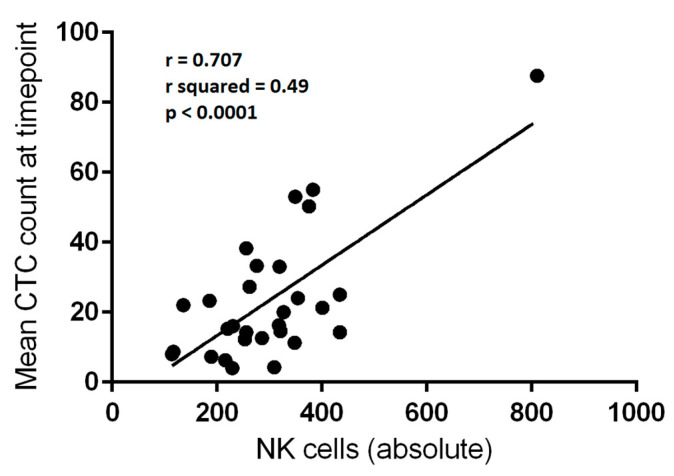
Correlation between absolute natural killer (NK-cell) count (per μL) and mean circulating tumour cell (CTC) count per 3 mL of blood at a given blood draw (*n* = 29). Linear regression, r = 0.707; r^2^ = 0.49; *p* < 0.0001.

The mean number of CTCs per 3 mL of blood from the 29 blood draw episodes which were subjected to flow cytometry analysis were compared with the absolute numbers of circulating lymphocytes. Linear regression analysis was employed for each variable. There was a significant correlation between mean CTCs per blood draw and both total lymphocyte count (*p* = 0.0258) and NK-cell count (*p* < 0.0001) (Table 2, Figure 1 and Figure 2). The correlation was relatively weak for total lymphocytes (r^2^ = 0.1709) but moderate for NK-cells (r^2^ = 0.4999).

### 3.4. Fractional Percentages of Lymphocytes Do Not Correlate with CTC Numbers

Analysis relating to the fractional percentage rather than absolute numbers of each lymphocyte subgroup was also performed. No significant correlation was evident between any lymphocyte fraction and the mean CTC count at that blood draw (Table 3).

### 3.5. Platelet Counts Weakly Correlate with CTC Numbers

**Figure 3:**
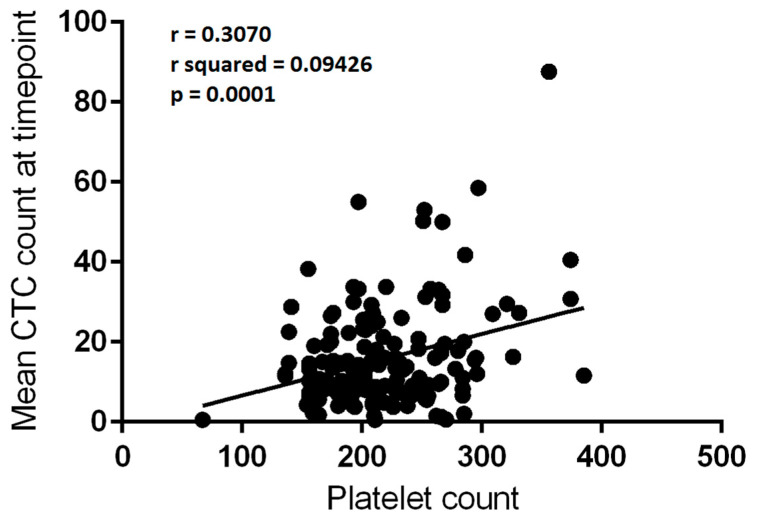
Correlation between platelet count (×10^9^/L) and mean circulating tumour cell (CTC) count per 3mL of blood at a given blood draw (*n* = 150). Linear regression, r = 0.3070; r^2^ = 0.09426; *p* = 0.0001.

There were 150 matched blood draw CTC count and platelet count values subjected to analysis. There was a significant correlation between platelet count and mean CTC count per 3 mL of blood at a given blood draw episode (r^2^ = 0.09426, F = 15.403, *p* = 0.0001—Figure 3). The low r^2^ value indicated that the correlation was relatively weak, and much of the variance in CTC count was not explained by the platelet count.

### 3.6. Absolute NK-Cell and Platelet Count Correlations with CTCs Are Independent of Each Other

Multiple regression analyses were performed to determine the relationships between CTC numbers and the flow-cytometry-derived lymphocyte subsets and platelet counts. There were 27 complete sets of data subjected to analysis. The absolute number of CD4+ T-lymphocytes (*p* = 0.0246), NK-cells (*p* = 0.0015) and platelets (*p* = 0.047) all independently correlated with the mean CTC count per blood draw. The CD8+ T-lymphocyte count and B-lymphocyte count did not (Table 4). CD3 expression was not included in the model due to multicollinearity, CD4+ and CD8+ cells being largely mutually exclusive subsets of the overall CD3+ T-cell population. Total lymphocytes were not included for the same reason. The adjusted R^2^ of the model with 21 degrees of freedom was 0.5526, indicating that more than half of the variation in CTC numbers could be predicted by the explanatory variables.

### 3.7. Subgroup Analysis of CTC/Platelet Count Correlations

#### 3.7.1. Platelet Counts Weakly Correlate with CTC Numbers in the Control Group, but Not the Exercise Group

**Figure 4:**
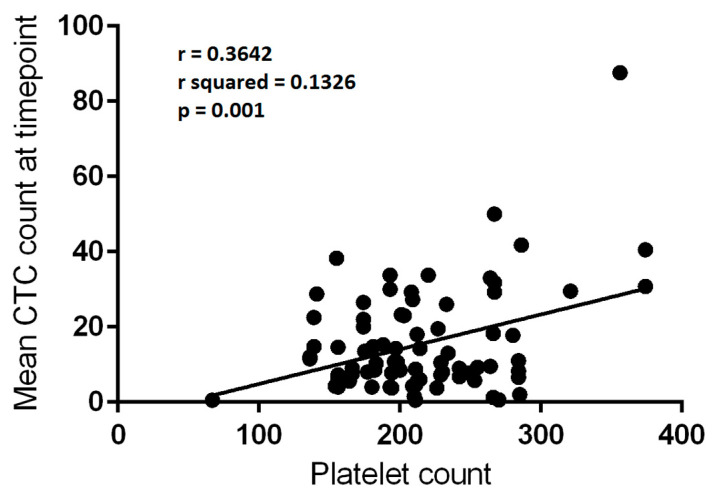
Correlation between platelet count (×10^9^/L) and mean circulating tumour cell (CTC) count per 3 mL of blood at a given blood draw in control group (*n* = 79). Linear regression, r = 0.3642; r^2^ = 0.1326; *p* = 0.001.

The CTC/platelet count correlation described in Section 3.5 persisted when the 79 measurements from the control group were analysed, although the correlation remained weak (r = 0.3642, r^2^ = 0.1326, F = 11.776, *p* = 0.001—Figure 4). There was a trend towards a correlation in the 71 measurements from the exercise group, but the finding did not reach statistical significance (r = 0.2166, F = 3.397, *p* = 0.0696).

#### 3.7.2. Platelet Counts Weakly Correlate with CTC Numbers in the Overweight/Obese Group, but Not the Normal Weight Group

A similar weak correlation was identified between platelet count and mean CTC count at a given blood draw episode within the 123 data pairs in the overweight/obese group (r = 0.3474, r^2^ = 0.1207, F = 16.611, *p* < 0.0001). There was no such identifiable correlation in the 27 data pairs of the normal weight group (r = 0.05369, F = 0.07226, *p* = 0.7903).

#### 3.7.3. Platelet Counts Weakly Correlate with CTC Numbers in Blood Draws Which Lacked Platelet Cloaking

**Figure 5:**
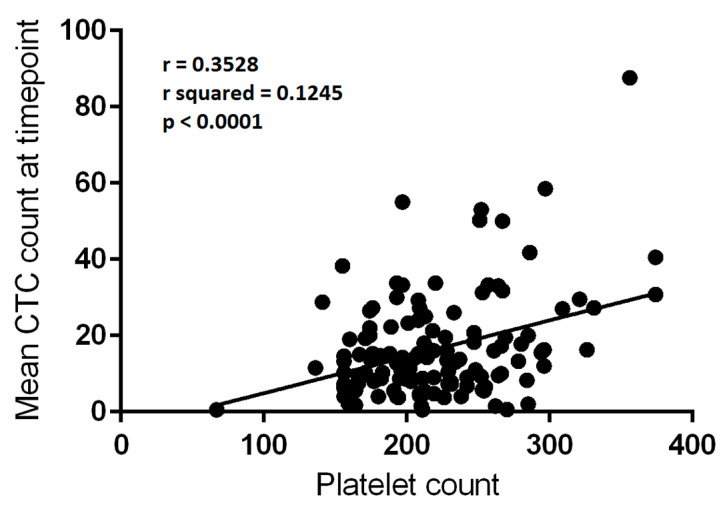
Correlation between platelet count (×10^9^/L) and mean circulating tumour cell (CTC) count per 3 mL of blood at a given blood draw which lacked platelet-cloaking (*n* = 128). Linear regression, r = 0.3528; r^2^ = 0.1245; *p* < 0.0001.

When the presence of platelet cloaking on any filter from a given blood draw was used to define subgroups, a weak correlation was present in the absence of platelet cloaking (r = 0.3528, r^2^ = 0.1245, F = 17.916, *p* < 0.0001, 128 data points—refer to Figure 5). The correlation was absent in the 22 blood draws in which platelet cloaking had been identified (r = −0.03812, F = 0.02911, *p* = 0.8662).

### 3.8. Subgroup Analysis of Flow Cytometry-Derived Lymphocyte Populations

#### 3.8.1. BMI Does Not Predict Differences in Circulating NK-Cell Numbers

When subgroups were considered, there was no significant difference in the percentage of circulating NK-cell numbers between the overweight/obese (17.65%) and normal weight (17%) groups (*p* = 0.7743, unpaired *t*-test). Comparison of absolute NK-cell numbers also did not demonstrate a significant difference (overweight/obese 299.7/μL, normal weight 302.5/μL, *p* = 0.5536, Mann–Whitney test).

#### 3.8.2. Exercise and Control Group Participants Had Significant Differences in Subsets of Circulating Lymphocytes

**Figure 6:**
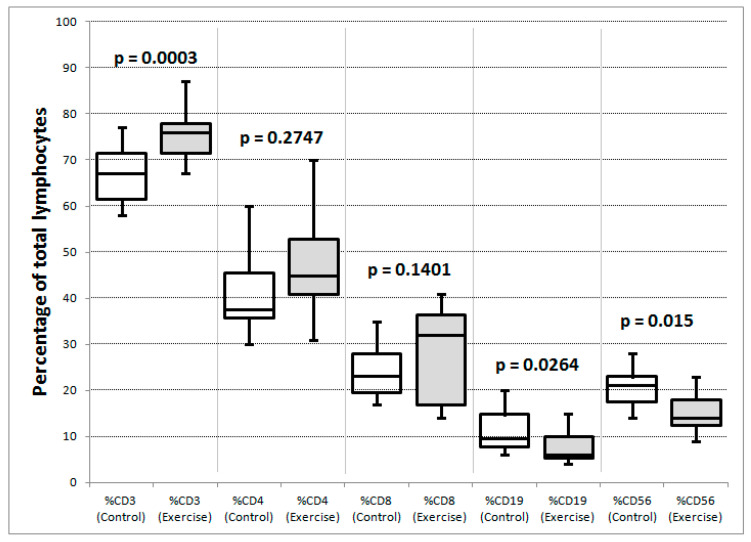
Comparison of lymphocyte subset percentages between control (white box plots) and exercise groups (shaded box plots). Maximum, minimum, quartiles and median are illustrated for each distribution. The *p* values are for unpaired *t*-tests (CD3, CD8, NK-cell) and Mann–Whitney tests (CD4, B-cell) depending on whether the data passed or failed a normality test.

**Figure 7:**
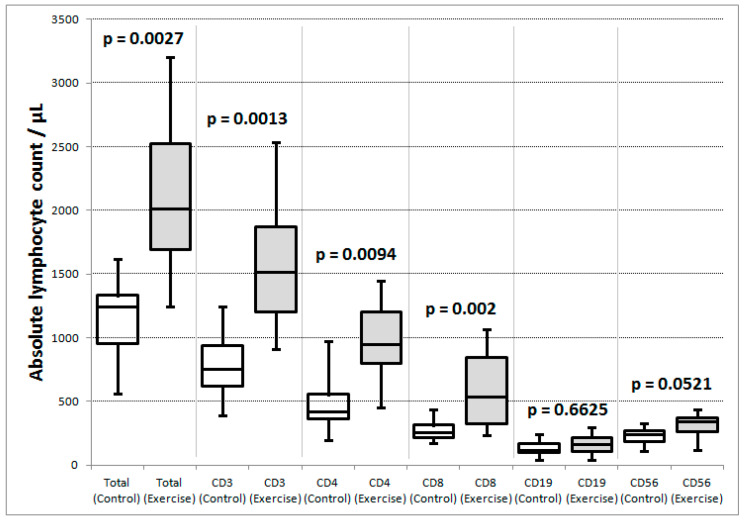
Comparison of absolute lymphocyte counts per μL between exercise (white box plots) and control groups (shaded box plots). Maximum, minimum, quartiles and median are illustrated for each distribution. The *p* values are for Mann–Whitney tests.

When the exercise and control groups were compared across all timepoints, the exercise group had significantly higher mean proportions of CD3+ T-lymphocytes (75.4 vs. 67%) and significantly lower proportions of B-lymphocytes (8 vs. 11.9%) and NK-cells (15.5 vs. 19.7%) than the control group, respectively, (see Table 5, Figure 6). There were significantly higher absolute counts in the exercise than control group (Figure 7) in total lymphocytes and all lymphocyte subgroups except for B-lymphocytes and NK-cells, which exhibited a strong trend towards significance: *p* = 0.0521.

## 4. Discussion

This study demonstrated positive correlations between CTC numbers (as defined by morphological criteria) and platelet count, total lymphocyte count and NK-cell count, in men with advanced PrCa who participated in the ExPeCT trial. By multiple regression analysis CD4+ T-cells, NK-cells and platelets independently correlated with CTC numbers. Correlation was strongest with absolute numbers of circulating NK-cells, and weaker with platelets and total lymphocytes. These findings suggested close interactions between the immune system and platelets relating to their responses to CTCs. A secondary finding identified differences in circulating lymphocyte subsets between ExPeCT participants who were randomized to an organized exercise programme, and those who were not.

A moderate correlation between CTC numbers and circulating NK-cells was detected in this study. A significant correlation was evident with absolute concentrations of lymphocytes, but not with percentages. The percentage of NK-cells in a given blood draw varied from 9 to 28% in the current study, but the interquartile range was only 16–22%. As such, 29 data points may not have provided sufficient statistical power to detect a true relationship over such a narrow range of percentages. There are few published studies describing correlations between lymphocyte subsets and CTCs. One study in inflammatory breast cancer found no correlation between CTC numbers and total lymphocyte count, but did find that patients with more CTCs “had significantly lower percentages of CD3+ T cells and TCR-activated CD8+ T cells that synthesized TNF-α and IFN-γ, and a higher percentage of T-regulatory lymphocytes” [18]. A study of 83 late-stage non-small cell lung cancer patients, which employed an antibody-dependent FISH methodology for enumerating CTCs, found that the percentages of CD3+, CD4+ and NK-cells were lower in CTC-positive than in CTC-negative patients [19]. Unlike in the current study, correlations with absolute numbers of the various lymphoid subpopulations were not reported. In treatment-naïve triple-negative breast cancer patients, there was a positive correlation between CTC status and peripheral NK-cell ratio, and in fact the combination of CTC count and NK-cell enumeration could predict progression free survival [20]. The correlation in the current study between CTC count and total lymphocytes was weak (R squared = 0.1709), whereas that with the NK-cell subset was stronger (R squared = 0.49). While absolute NK-cell numbers and percentages generally appear to be increased in advanced cancer, the functional ability of circulating NK-cells to lyse CTCs may be impaired. Using a chromium-51 percent specific lysis assay patients with breast cancer were found to have significantly decreased responses by their immune cells when they had more than 5 CTCs identified per 7.5 mL of blood by the CellSearch^®^ system [21]. A study involving patients with breast, colorectal and PrCa found higher proportions of circulating NK-cells in the peripheral blood compared to healthy controls, and also that patients with high numbers of CellSearch^®^-detected CTCs had impaired cytolytic activity compared to controls [22]. Functional NK-cell subsets can be defined through relative surface expression of CD56 and CD16, the receptor for FcγRIII. NK-cell subsets with less anticancer activity, particularly CD56^high^CD16+ and CD56^low^CD16− subsets, are increased in patients with advanced breast cancer [23]. Whether this is linked to the impairment of NK-cell function by platelets previously reported remains to be elucidated [11,12,13,14]. The flow cytometry assay employed in the current study detected but did not distinguish between populations of CD56^high^CD16+, CD56^high^CD16− and CD56^low^CD16+ NK-cells, and so it is unclear whether the increased NK-cell numbers associated with increased CTC counts in PrCa have a similar reduction in anticancer activity to those in advanced breast cancer. Further analysis of functional NK-cell subsets in PrCa would be of benefit in this regard. In the current study a positive (but relatively weak) correlation was identified between platelet count and mean CTC numbers per filter at a given blood draw. Previously, a study from Tibet compared platelet counts in CTC-positive and CTC-negative patients with lung cancer, and found significantly higher platelet counts in CTC-positive participants [24]. A study of oesophageal squamous cell carcinoma patients using both the ISET system (similar to ScreenCell^®^) and CellSearch^®^, had similar findings [25]. Although CTC enumeration was performed in the Li study for ISET/CellSearch^®^ comparison purposes, correlation between platelet counts and CTC numbers as a continuous variable was not available in these studies. To our knowledge, the current study is the first to establish a relationship between CTC numbers and platelet counts in patients with PrCa. There is no reason to assume that such a relationship would not exist in epithelial malignancies other than gastric and prostatic adenocarcinoma, but further research is required in this regard. When subgroups were analysed, the CTC-platelet correlation persisted in the control group and the group that was overweight at randomization, and there was a non-significant trend towards the correlation in the exercise group. However there was no demonstrable correlation in the group that had normal weight at randomization. This may be due to lack of statistical power because only 27 data pairs were available for assessment. If, however, there truly were no correlation in this group, it would suggest that whatever mechanism drove the relationship between the platelets and CTC numbers was attenuated among the (minority) subpopulation of patients of normal weight. The platelet/CTC correlation was demonstrated in those blood draws where platelet cloaking had not been identified, but not in the blood draws where at least one cloaked CTC was observed. This may have been because overweight men have greater numbers of circulating platelets and consequently a greater tendency for platelets to cloak their CTCs. While it was difficult for technical reasons to demonstrate and measure platelet cloaking directly, the presence and absence of a direct correlation between CTCs and circulating platelet numbers in men who were overweight and of normal weight, respectively, did provide supportive evidence indicating a central role in obesity in platelet-CTC interactions, which merits further investigation.

Several clinical inflammation-based indicators, known to be associated with worse outcomes in many carcinomas, include an assessment of platelet counts. For example Zheng et al. [26] found significant correlations between the systemic immune-inflammation index and CTC count, and between the platelet–lymphocyte ratio and CTC count in gastric cancer patients. The use of these systemic inflammation-based composite indicators is attractive as we hypothesise that platelet-CTC interactions are intimately associated with and dependent upon systemic inflammation, which can, in turn, be driven by the metabolic abnormalities found in obesity. A clinical trial attempting to assess the change in CTC numbers in breast cancer patients through the inhibition of platelet function with clopidogrel and aspirin was unsuccessful [27]. This trial was impaired by the low baseline level of CTCs in the trial participants, and might have been more successful with a cohort of patients in whom CTCs were abundant, such as those in the ExPeCT trial.

In the current study, flow cytometry demonstrated significantly more circulating total lymphocytes in the exercise group, driven predominantly by a proportionate increase in CD4+ and CD8+ T-lymphocytes. The absence of a significant corresponding increase in B-lymphocytes and NK-cells caused the proportions of these populations relative to T-lymphocytes to appear lower. In the acute setting, it is well recognised that strenuous exercise in healthy subjects produces transient lymphocytosis [28,29], including increased absolute numbers of CD4+ and CD8+ T-lymphocytes as well as B-lymphocytes and NK-cells [30]. In acute exercise, NK-cell numbers in the blood increase rapidly (within two minutes), reach a maximum within 30 min, and remain elevated during exercise for up to three hours [31]. In the current study, blood samples were taken at clinic visits rather than during or after exercise episodes, so any acute increases in circulating NK-cell numbers would be expected to have returned to normal by the time of sample acquisition. Nonetheless, given the established role of NK-cell mediated cytotoxicity in cancers, it is attractive to consider evidence of NK-cell recruitment as a likely mechanism whereby exercise can improve cancer outcomes. In a mouse model, exercise with associated IL6-mediated NK-cell recruitment is associated with reduced tumour growth [32]. In clinical studies NK-cell numbers increased post-exercise in breast cancer survivors although to a lesser extent than in healthy controls [33]. In chronic exercise, many studies of healthy young controls showed increased cytotoxic activity of circulating NK-cells [34], but generally no change in absolute numbers. The effect of increased cytotoxicity in older adults is less pronounced [35]. No change in numbers of lymphocyte subsets was demonstrated over the “chronic” timescale of the exercise intervention in the current study.

There are some limitations associated with this study. The morphological criteria for CTC identification, while widely used [36,37], are not entirely specific and no other validatory assay was available. Platelet cloaking of CTCs proved difficult to assess in practice, as many CTCs were present on the ScreenCell^®^ filters as bare nuclei lacking cytoplasm—a feature which may be due to shear forces experienced by the CTCs during vacuum-induced filter preparation.

## 5. Conclusions

Independent correlations were demonstrated in advanced PrCa between CTC numbers and NK-cell counts and platelet count. The demonstration of a relationship to the platelet count is the first such report in men with PrCa. Although correlation does not imply causation it is clear from the literature that CTCs interact with platelets in various ways to enhance their metastatic potential, and our findings provide further evidence of an association between the two. In PrCa patients, as in most cancers (with the exception of brain tumours and critically located head and neck carcinomas), metastatic disease is the most frequent cause of cancer-related death. The therapeutic implications are profound as if there were a way to break the partnership between the villainous CTC and its nefarious platelet sidekick, then there would be potential for a new type of anticancer drug specifically targeted at this crucial step in the metastatic cascade. The correlations with various lymphocyte subsets are also interesting and emphasise the complex nature of interactions between CTCs and the innate and adaptive immune systems. Overall this study provides useful support for the hypothesis that metastasis, platelet function, systemic inflammation and hypercoagulability are closely linked in advanced cancer, and it elucidates several potentially valuable directions for future research.

## Figures and Tables

**Table 1:** Categorisation of ScreenCell filters and blood draw episodes.

Category	Filters (*n* = 598)	% of Total	Blood Draws (*n* = 162)	% of Total
T0	220	36.8%	61	37.6%
T3	197	32.9%	52	32.1%
T6	181	30.3%	49	30.3%
Exercise group	290	48.5%	78	48.1%
Control group	308	51.5%	84	51.9%
Exposed (BMI ≥ 25)	491	82.1%	133	82.1%
Non-exposed (BMI < 25)	107	17.9%	29	17.9%
CTCs absent	31	5.2%	0	0%
CTCs present	567	94.8%	162	100%
Platelet cloaking absent	565	94.5%	138	85.2%
Platelet cloaking present	33	5.5%	24	14.8%

**Table 2:** CTC number/absolute lymphocyte count correlations (linear regression analysis). Bold is used here to highlight significant values (*p* < 0.05).

Category	r	r^2^	F	*p*
Total lymphocyte count	0.4134	0.1709	5.566	**0.0258**
Absolute CD3 count	0.3122	0.09745	2.915	0.0992
Absolute CD4 count	0.2751	0.07566	2.21	0.1487
Absolute CD8 count	0.2372	0.05628	1.61	0.2153
Absolute B-cell count	0.2292	0.05254	1.497	0.2317
Absolute NK-cell count	0.707	0.4999	26.987	**<0.0001**
CD4–CD8 ratio	−0.00515	0.0000265	0.000715	0.9789

**Table 3:** CTC-number/lymphocyte fraction correlations ^1^.

Lymphocyte Subset	r	r^2^	F	*p*
% CD3	−0.1893	0.03582	0.1587	0.6935
% CD4	−0.1749	0.03058	0.8518	0.3642
% CD8	0.09642	0.009296	0.2533	0.6188
% B-cell	−0.07644	0.005842	0.1587	0.6935
% NK-cell	0.2478	0.06143	1.767	0.1949

^1^ Linear regression analysis.

**Table 4:** Multiple regression analysis of mean circulating tumour cell (CTC) count per blood draw against absolute lymphocyte and platelet counts. CI: confidence interval. SE: standard error. Bold highlights signficant values (*p* < 0.05).

Variable	Coefficient	SE ^1^	95% CI ^2^ Lower	95% CI ^2^ Upper	*t* Ratio	*p* =
(constant)	−26.445	12.256	−51.937	−0.9525	2.158	**0.0427**
CD4	−0.02426	0.01002	−0.0451	−0.003425	2.422	**0.0246**
CD8	0.00635	0.01089	−0.0163	0.029	0.583	0.5661
B-cell	0.03094	0.03044	−0.03237	0.09424	1.016	0.321
NK-cell	0.09942	0.02726	0.04273	0.1561	3.648	**0.0015**
Platelets	0.1555	0.07365	0.002259	0.3086	2.111	**0.047**
R squared = 0.6386, adjusted R squared = 0.5526, Multiple R = 0.7992, F = 7.4229						
Degrees of freedom = 21						
***p* = 0.0004**						

^1^ SE: Standard error. ^2^ CI: Confidence interval.

**Table 5:** Comparison of mean lymphocyte counts between exercise and control groups. Bold highlights significant values (*p* < 0.05).

Lymphocyte Subset	Control	Exercise	*p* =	Test
% CD3	67%	75.4%	**0.0003**	Unpaired *t*
% CD4	43.5%	47.1%	0.2747	Mann–Whitney
% CD8	22.9%	27.9%	0.1401	Unpaired *t*
% B-cell	11.9%	8%	**0.0264**	Mann–Whitney
% NK-cell	19.7%	15.5%	**0.015**	Unpaired *t*
Absolute ^1^ CD3	987.6	1599.7	**0.0013**	Mann–Whitney
Absolute CD4	680	986.4	**0.0094**	Mann–Whitney
Absolute CD8	293.7	606.7	**0.002**	Mann–Whitney
Absolute B-cell	182.4	167.3	0.6625	Mann–Whitney
Absolute NK-cell	280.7	318.5	0.0521	Mann–Whitney
Total lymphocytes	1468.1	2111.5	**0.0027**	Mann–Whitney
CD4:CD8 ratio	2.272	2.075	0.6467	Mann–Whitney

^1^ Absolute lymphocyte counts per mL.

## Data Availability

There are ethical restrictions to the sharing of this de-identified dataset. Informed consent for use of the data does not allow for sharing individual data publicly. Trinity College Dublin is responsible for clinical data and the data protection officer can be contacted at evelyn.fox@tcd.ie for all future data requests. The contact details for each ethics committee are as follows: NRES Committee London—Camden and Islington (nrescommittee.londoncamdenislington@nhs.net), The Mater Misericordia Hospital Research Ethics Committee, Dublin (mmh@mater.ie), Beaumont Hospital Ethics, Dublin (beaumontethics@rcsi.com), SJH/AMNCH Research Ethics Committee, Dublin (researchethics@tuh.ie) and St Luke’s Radiation Oncology Network, Dublin (radiotherapy.stlukes@slh.ie).

## Appendix A

The flow-cytometry-derived lymphoid cell numbers were subjected to ANOVA analysis to compare levels across timepoints for the nine participants for whom T0, T3 and T6 samples were available. No significant variance was identified between the T0, T3 and T6 samples in total lymphocytes (*p* = 0.9712, Friedman test), absolute CD56+ (NK-cell) count (*p* = 0.898, ordinary ANOVA) or any other lymphocyte group (Table A1, Figure A1 and Figure A2).

**Table A1:** ANOVA of flow-cytometry derived lymphocyte counts per timepoint.

Lymphocyte Subsets	*p* =	F = (Fr =)	Effective Matching, *p* =	Matching F =	Test
% CD3	0.9131	0.182 (Fr)	0.0003	7.504	Friedman test
% CD4	0.8485	0.166	<0.0001	19.483	Repeated measures ANOVA
% CD8	0.1814	1.903	<0.0001	1.903	Repeated measures ANOVA
% B-cell	0.5109	0.7005	<0.0001	10.51	Repeated measures ANOVA
% NK-cell	0.9466	0.05503	0.0137	3.62	Repeated measures ANOVA
Absolute CD3	0.569	1.556 (Fr)			Friedman test
Absolute CD4	0.6801	0.395	<0.0001	16.104	Repeated measures ANOVA
Absolute CD8	0.6854	0.889 (Fr)			Friedman test
Absolute B-cell	0.8716	0.1386	0.0269	3.068	Repeated measures ANOVA
Absolute NK-cell	0.898				Ordinary ANOVA
Total lymphocytes	0.9712	0.222 (Fr)			Friedman test
CD4–CD8 ratio	0.4481	0.8443	<0.0001	77.735	Repeated measures ANOVA
